# Cortical-Subcortical Interactions in Depression: From Animal Models to Human Psychopathology

**DOI:** 10.3389/fnsys.2016.00020

**Published:** 2016-03-07

**Authors:** Aaron S. Heller

**Affiliations:** Department of Psychology, University of MiamiCoral Gables, FL, USA

**Keywords:** depression, emotion, connectivity, animal models of mental disorders, clinical neuroscience

## Abstract

Depression is a debilitating disorder causing significant societal and personal suffering. Improvements in identification of major depressive disorder (MDD) and its treatment are essential to reduce its toll. Recent developments in rodent models of MDD and neuroimaging of humans suffering from the disorder provide avenues through which gains can be made towards reducing its burden. In this review, new findings, integrating across rodent models and human imaging are highlighted that have yielded new insights towards a basic understanding of the disorder. In particular, this review focuses on cortical-subcortical interactions underlying the pathophysiology of MDD. In particular, evidence is accruing that dysfunction in prefrontal-subcortical circuits including the amygdala, ventral striatum (VS), hippocampus and dorsal raphe nucleus (DRN) are associated with MDD status.

## Introduction

According to the most recent Global Burden of Disease Study sponsored by the World Health Organization, Harvard School of Public Health, and the World Bank, major depressive disorder (MDD) is the second leading cause of disability worldwide (Moussavi et al., 2007; World Health Organization, 2009). MDD, defined by a constellation of signs and symptoms including alterations in mood, hedonic capacity, appetite, sleep, energy, and cognition, is a common disorder affecting 21% of women and 11% of men in the USA in their lifetime (Kessler et al., 2003). MDD also has high rates of comorbidity with other mental illness such that nearly three-quarters of people who meet the criteria for depression during their lifetime will also suffer from another psychiatric disorder. More specifically, approximately three-fifths will be co-morbid for an anxiety disorder, one-quarter for substance-use disorders and one-third for impulse-control disorders (Rush et al., 2005). It is associated with severe morbidity resulting in lost workdays and over utilization of health care services. Patients with MDD also exhibit a markedly shortened lifespan, not only due to suicide, but to a well-documented increased risk for other major medical disorders including coronary artery disease, diabetes, and certain cancers (Cowles et al., 2010). Moreover, it is now well established that medically ill patients with comorbid MDD experience significantly worse clinical courses for their medical illnesses, i.e., cancer, heart and kidney disease, etc., despite receiving standard medical treatment (Mavrides and Nemeroff, 2015). These findings, taken together with the more than 40,000 suicides in the USA (and one million worldwide), demonstrate the massive public health problem represented by MDD. It is now clear that the longer depressive symptoms persist, the less likely patients are to respond optimally to treatment, with the unfavorable consequence of persistent morbidity and increased mortality (Posse and Nemeroff, 2012). In sum, the effects of an MDD diagnosis are vast and demand a wide lens to grasp the full impact it can have. As such, a greater understanding of the pathophysiology of MDD—both in animal models as well as in humans—are highly relevant to better understanding the disorder and to potentially improving treatment. The growing scientific and social awareness devoted to MDD is underscored by a recent special issue in Nature highlighting the disorder (2014a,b; Monteggia et al., 2014; Smith, 2014).

Despite a large and ever growing literature on the neural predictors of MDD course, this article highlights recent areas of particular growth and particularly on empirical evidence examining connectivity in untreated depressed patients. After a brief introduction to a variety of animal models of MDD, this article is organized by cortical-subcortical circuit and integrates both nonhuman and human data within each section. The circuits examined include prefrontal cortex (PFC)-ventral striatum (VS), PFC-amygdala, and PFC-dorsal raphe nucleus (DRN) along with brief descriptions of others. This article will end with brief considerations going forward.

It should be noted that while in this article the separate sections pertaining to cortical-subcortical circuits generally encompass the PFC as a whole, the PFC is clearly not a unitary region. The PFC is well-known to have subregions, and these subregions have differential roles in cognition (Badre, 2008), emotional regulation (Ochsner et al., 2012) and MDD (Koenigs and Grafman, 2009). It is the medial prefrontal cortex (mPFC) that has direct, monsynaptic connections to the VS and amygdala and is the area most typically examined in animal studies. Some imaging studies have found significant effects of connectivity between the DLPFC and subcortical structures, although these effects are likely mediated through an intermediary region as there are minimal direct projections between the DLPFC and subcortex.

## Animal Models of Depression

Several distinct animal models of MDD have been developed to examine both the pathophysiology of the disorders as well as to better understand the potential mechanisms behind treatment response. While an imperfect model to encapsulate the complexity and full diversity of symptoms experienced in human MDD (Krishnan and Nestler, 2010; Nestler and Hyman, 2010), animal models of MDD have aided our mechanistic understandings of the course of the disorder. Some rodent models of MDD include learned helplessness, social isolation/withdrawal, as well as the chronic social defeat stress (Krishnan and Nestler, 2010; Bagot et al., 2014) although there are several others (Ménard et al., 2015), many of which are not covered here.

In the learned helplessness paradigm, rodents are first conditioned to a repeated and uncontrollable stressor (e.g., a footshock). After several iterations of this uncontrollable stressor, rodents are given the opportunity to escape from a similar stressor but typically do not do so, hence the term “learned helplessness”. This behavioral decrement is reversed by SSRI administration (Petty et al., 1997; Zazpe et al., 2007). As such, it is defined as a deficit to escape an avoidable aversive stimulus and is measured by the degree of passivity in rodent. In rats exposed to the learned helplessness paradigm, changes in depressive behaviors (i.e., a lack of escape attempts) are closely related to persistent remodeling of hippocampal spines and synapses (Hajszan et al., 2009) and to mPFC regulation of subcortical structures.

In the chronic social defeat stress paradigm, a young mouse is placed in a cage with a larger adult male mouse and is subjected to brief periods of aggression by the larger mouse. After a series of bouts of these experiences, the younger mouse exhibits a variety of symptoms which appear to model human MDD including reduced locomotion (psychomotor retardation), decreased socialization, as well as suppressed interest in previously pleasurable activities (anhedonia; Duman and Monteggia, 2006; American Psychiatric Association, 2013). In the chronic social defeat stress paradigm chronic, but not acute treatment with selective serotonin reuptake inhibitors (SSRIs) normalizes many of the behavioral symptoms in this animal model. This is an important feature of the social defeat stress model because this time course of treatment response mimics the course of effective antidepressant treatment in humans (Krishnan and Nestler, 2008).

The social defeat stress model also appears to cause specific alterations in cortical-subcortical interactions. In particular, interactions between striatal, amygdalar and medial frontal regions are affected by social defeat stress (Tye and Deisseroth, 2012; Russo and Nestler, 2013). The chronic social defeat stress paradigm is caused by placing a rodent in the same cage with a much larger rodent. The smaller rodent acts in a similar helpless way and a depression-like phenotype characterized by anhedonia (as evidenced by decreased sucrose intake) and social avoidance behaviors is engendered (Ménard et al., 2015). Importantly, these behaviors can be reversed by chronic treatment with antidepressants. The requirement for chronic antidepressant treatment models the timecourse of antidepressant effects in humans and adds face validity to the chronic social defeat stress paradigm.

## PFC-VS Connectivity

Interactions between the PFC and VS make up a primary part of the reward circuit (Haber and Knutson, 2010) and has been implicated in the pathophysiology of MDD (Admon and Pizzagalli, 2015a; Heshmati and Russo, 2015). Initial studies in humans with degeneration or lesions of the basal ganglia found that these patients were at higher risk for developing major depressive episodes (Price and Drevets, 2012). More recently, deep brain stimulation (DBS) of the VS/Ventral Capsule appears to successfully reduce depressive symptomatology in treatment resistant MDD (Malone et al., 2009) implicating parts of this network in MDD.

In addition, there are dense dopaminergic projections from the VTA to the VS and these projections are implicated in reward learning, and in particular the reward prediction error (Schultz, 2015). The mPFC contains dense projections to the VS generally and the nucleus accumbens (NAc) in particular. These projections are glutamatergic in nature and primarily terminate onto GABAergic medium spiny neurons within the VS (Haber and Knutson, 2010). Thus, it has been suggested that the mPFC can exert a regulatory effect on the VS, although it should be noted that several findings have also suggested for a separate role for the mPFC in coding the value of stimuli as well (Grabenhorst and Rolls, 2011). Disentangling the specific computational roles for the mPFC in regulation and coding of value is an ongoing active area of research.

### Animal Models

A growing literature using animal models of depression has investigated the role of PFC-VS circuits. High-frequency optogenetic stimulation of medial prefrontal cortical glutamatergic afferents increase c-Fos expression in the NAc and reverse social avoidance behavior after chronic social defeat stress (Covington et al., 2010). Using optogenetics, activation of mPFC terminals in the NAc has been shown to elicit anti-depressant like effects following social defeat stress (Vialou et al., 2014; Christoffel et al., 2015). However, it is unclear whether the stimulation is selective to the PFC terminals on the NAc or something more specific to the local circuit dynamics. For example, lower frequency stimulation of PFC to NAc terminals or silencing does not affect social avoidance (Christoffel et al., 2015) and it appears that which mPFC subregions are being stimulated are essential to the downstream behavioral effects (Warden et al., 2012). For example, when stimulating a subregion of the mPFC that projected to the DRN—the major source of serotonin to the forebrain—immobility in the forced swim test decreased.

### Task Based Human Imaging Studies

Human imaging has frequently, though not consistently, demonstrated abnormalities in PFC-VS connectivity in MDD (Heller et al., 2009). Some discrepancies may be due to task or sample type and it has been suggested that examining distinct features of reward process may be helpful in illuminating under what specific conditions are PFC-VS dysfunction present (Salamone et al., 2007; Treadway and Zald, 2013).

In a recent study using a naturalistic positive mood induction, Admon and Pizzagalli (2015b) found that patients with MDD sustained positive emotion for a shorter duration as compared with healthy controls and that this was associated with decreased effective mPFC-VS connectivity using dynamic causal modeling. In particular, before the mood induction, controls and depressed patients both displayed similar mPFC-to-VS connectivity. However, following the mood induction, controls demonstrated a shift of effective connectivity towards a more reciprocal relationship (i.e., mPFC-to-VS as well as VS-to-mPFC) whereas depressed patients evidenced an unchanging—mPFC-to-VS connectivity without any reciprocal connectivity emerging. Similarly, in a reappraisal study of adults with MDD (Heller et al., 2009), we showed that depressed patients relative to healthy volunteers had more rapid habituation of VS activity when upregulating (i.e., increasing) positive emotion. Depressed patients also displayed more rapid decoupling of VS-DLPFC connectivity compared with healthy controls when increasing positive emotion in response to viewing positively valenced images. The degree of habituation in VS activity correlated with self-reported positive affect in daily life with depressed patients who reported higher levels of positive affect showing less VS habituation. These studies suggest that the temporal dynamics of PFC-VS connectivity may be impaired in MDD and in particular the ability to maintain adaptive functional interactions in the face of appetitive stimuli.

Other studies using distinct sets of stimuli have found similar results. One study using a passive picture-viewing task found decreased ACC-striatal connectivity in depressed patients as compared with healthy controls when viewing positive pictures (Anand et al., 2005a). Another study using monetary gains found that depressed individuals show reduced connectivity between the dorsal anterior cingulate cortex (dACC) and dorsal striatum (Admon et al., 2015), perhaps reflecting abnormalities in the learning of reward contingencies (Niznikiewicz and Delgado, 2011) to behavioral outputs as well.

Studies have also examined PFC-VS connectivity abnormalities in a depressed aging sample. Using a probabilistic reversal learning task in older depressed patients, it was found that MDD status predicted aberrant vlPFC-striatal connectivity in response to unexpected reward (Dombrovski et al., 2015; i.e., a positive prediction error). It should be noted, however, that the striatal seed used in this study primarily included the putamen and not the ventral portion of the striatum that includes the NAc.

Overall, many of these studies suggest abnormalities in PFC-VS functioning, particularly in response to appetitive stimuli. The majority of these studies have used either images or financial rewards. The magnitude of hedonic effect in response to these stimuli can be somewhat limited, however, which may limit researchers ability to examine the full spectrum of individual variation. As such, it will be worth considering how to best evoke hedonic anticipation and responses in MDD (Coan and Allen, 2007).

### Resting State Human Imaging Studies

While perhaps less theoretically constrained than task-based studies, one clear benefit of RSFC studies is the ease of repeatability and replication across samples and sites. It has been suggested that resting state connectivity abnormalities may reflect dysregulated self-representation in MDD (Sheline et al., 2010; Northoff et al., 2011; Marchetti et al., 2012). A recent meta-analysis found overall frontoparietal hypoconnectivity in MDD (Kaiser et al., 2015). Others have found an overall increase in default mode network connectivity in MDD (for recent reviews, see Dutta et al., 2014; Dichter et al., 2015).

While the majority of resting state studies examining abnormalities in MDD has focused on the default mode network, a growing literature has specifically examined cortical-subcortical interactions. Some RSFC studies examining VS connectivity have found decreased vmPFC-VS and sgACC- VS connectivity in adult depressed patients as compared with healthy controls (Furman et al., 2011; Kaiser et al., 2015) as well as in adolescents with MDD (Ho et al., 2015). However, another study found just the opposite in adolescents (Gabbay et al., 2013). RSFC between the VS and PFC is associated with the number of depressive episodes (Meng et al., 2014), as well as MDD severity (Satterthwaite et al., 2015b), suggesting that the topology of the striatum’s connectivity is associated with the course of episodes in MDD. In children and adolescents at high familial risk for MDD it was recently found that they had significantly decreased connectivity between the VS and other areas of the DMN as compared with a low risk group (Frost Bellgowan et al., 2015). Despite these positive findings, it is difficult to know the number of negative PFC-VS findings in RSFC studies of MDD, though it appears that there may be several (Dutta et al., 2014; Dichter et al., 2015).

### Structural Studies

A recent meta-analysis of studies using DTI in MDD found no specific abnormalities in the major white matter pathways connecting the PFC and VS (the medial forebrain bundle) and some empirical evidence (Guo et al., 2012a,b) to the contrary. Interestingly, however, a few recent studies have found that type of MDD may be associated with whether abnormalities in the medial forebrain bundle are present in depressed patients (Bracht et al., 2015b). In particular, it has been reported that individuals with melancholic MDD may be the ones who have specifically abnormalities in these white matter pathways, whereas nonmelancholic depressed patients appear not to have these abnormalitites (Bracht et al., 2014, 2015a).

## Prefrontal-Amygdala Connectivity

Experiments in rats, cats, and nonhuman primates have shown that the basal and lateral amygdala have reciprocal connections to the mPFC and, to a lesser extent, to the orbital PFC (Price, 2003). The amygdala is reciprocally connected to the PFC via the uncinate fasciculus (Von Der Heide et al., 2013). Further, the central and medial nuclei of the amygdala have outputs to the ventromedial striatum, hypothalamic and brainstem areas and these glutamatergic outputs are directly involved in motivation and visceral control (Price, 2003; Stuber et al., 2011). In MDD, it has been suggested that there is a reduction of amygdala-mPFC (including the rostral ACC and sgACC) connectivity in response to emotional stimuli and that this hypoconnectivity normalizes with treatment (Phillips et al., 2015).

### Animal Models

Much of the work examining PFC-amygdala connectivity using optogenetics in rodents has done so using a rodent anxiety model (Tye and Deisseroth, 2012). In these studies, the degree of specificity to anxiety-like behaviors is not entirely clear, although some specific models of MDD, including the chronic stress model increases spine number and dendritic complexity within the basolateral subregion of the amygdala (Vyas et al., 2006) which has been seen in rodent models of MDD (Ménard et al., 2015). Distinct behavioral effects of optogenetic stimulation within the amygdala have been observed when either basolateral amygdala (BLA) neurons or their projections to the central amygdala (CeA) have been modulated. Activation of BLA neurons with projections toward the lateral aspect of the CeA have been found to be anxiolytic, whereas activation of other BLA neurons cause an increase in anxiety (Tye et al., 2011).

Relatedly, in nonhuman primates, ablation of pathways connecting the OFC to the amygdala leads to an inability to update the value previously assigned to a specific stimulus (Murray et al., 2011). One hypothesis that has been put forth regarding MDD is that abnormalities in this ventral PFC-amygdala circuit may underlie a overly negative representation of the self and impede the ability to update these representations towards more positive and realistic representations (Murray et al., 2011; Robinson et al., 2012).

### Task Based Human Imaging Studies

In two separate studies, and in response to happy faces, Almeida et al. (2009, 2011) have found reduced (near zero) effective connectivity in specifically left sided vmPFC-amygdala. These effective connectivity abnormalities appeared to be specifically in response to happy faces and were present primarily for females with MDD as compared with healthy controls. These studies also demonstrated a dissociation in subregions of the PFC such that while OFC- and vmPFC-amygdala connectivity appear to mediate associations between neural responses to happy faces and MDD status, sgACC-amygdala connectivity appeared to differentiate groups in response to fear faces. That said, a formal statistical analysis testing whether connectivity values were significantly different in these conditions was not performed, and makes inferring the degree of specificity difficult.

An interesting and recent study compared neural responses in four groups: a depressed group, a non-depressed group with family history of MDD, a sample of remitted depressed individuals as well as a healthy control group. In this study, Young et al. (2016) used fMRI to examine the neural mechanisms underlying autobiographical recall of positive and negative memories. While potentially less well standardized than using financial rewards or visual images, recollection of autobiographical memories may be more effective at inducing an emotional state. Currently depressed patients showed decreased dACC-amygdala and medial frontopolar cortex-amygdala connectivity when recalling positive memories as compared with all other groups. In contrast, when recalling negative memories, depressed patients showed increased amygdala-dACC connectivity as compared with the other groups, but once again attenuated medial frontopolar-amygdala connectivity as compared with the other groups. It should be noted that there are no direct anatomical projections between anterior frontopolar cortex and the amygdala. This raises the question as to what region might be the intermediate weigh-station supporting this finding. Nonetheless, these findings suggest abnormal reduction in frontopolar-amygdalar connectivity during autobiographical recall regardless of valence, and that abnormalities in ACC-amygdala connectivity may be present (or not) depending on context and valence.

A somewhat earlier study also compared multiple patients (Etkin and Schatzberg, 2011). In this study, patients participants included: patients with MDD, with generalized anxiety, a group with comorbid MDD and anxiety as well as a healthy control group. These individuals participated in an emotional incongruence task. In this task, participants are presented with emotional faces upon which affective words are overlaid. These emotional words are either congruent or incongruent with the emotion presented on the face. Participants categorize the emotion presented on the face. Etkin and Schatzberg (2011) found that in the incongruent condition—when the word and face did not cohere, patient groups demonstrated heightened (i.e., less negative) sgACC-amygdala connectivity. Given the incongruency of the stimuli and the need to overcome a stroop-like prepotent response, these results were interpreted not in the vein of affective responses *per se*, but as abnormalities in the regulation of affective responses.

In another study of adult depressed patients, Carballedo et al. (2011) used a blocked design in which depressed patients were presented with emotional (sad and angry) faces. In response to these emotional faces, adult patients with MDD evidenced decreased bilateral OFC-amygdala connectivity as well as decreased unilateral right ACC-amygdala connectivity as compared with healthy controls (Carballedo et al., 2011). Similarly, in a similar task in which depressed patients were presented with sad faces, Chen et al. (2008) found decreased amygdala-IFG and amygdala-mPFC connectivity in depressed patients as compared to healthy controls.

Using an implicit fear task in which depressed adolescents were presented with faces showing differing degrees of fearfulness while having to solely identify the gender of the face, Ho et al. (2014) found that depressed adolescents showed increased sgACC-amygdala connectivity as compared with healthy controls. It should be noted, however, that the amygdala cluster reported in the study and used as a seed in the connectivity analyses extended into the ventral portion of the striatum. Mothers with postpartum MDD appear to show similar effects. In a blocked design fMRI task looking at the neural response to negative emotional faces, mothers with postpartum MDD evidenced less dmPFC-amygdala connectivity as compared to nondepressed mothers (Moses-Kolko et al., 2010).

Taken together, the majority of task-based fMRI studies looking at PFC-amygdala connectivity have used passive processing of emotional faces. In general, decreased PFC-amygdala connectivity has been reported in these studies. Several areas have been described as showing aberrant connectivity, including mPFC, anterior mPFC, OFC, sgACC and dACC. Whether each of these PFC areas has distinct roles in the processing or regulation of amygdala activity remains to be seen and more precise delineation of these unique contributions is critical. Further, from many of these studies it is impossible to know whether this deficit is due to affective processing or affective regulation. Moreover, in tasks that use the presentation of emotional faces it is difficult to know the degree to which any abnormalities are in the experience of emotion itself or in the external processing of valenced stimuli. The continued parsing of affective perception, experience and regulation is critical to disentangle the role of these networks in the pathophysiology of MDD.

### Resting State Human Imaging Studies

Overall, recent RSFC meta-analyses and reviews have generally found hypoconnectivity between the mPFC (as well as other nodes of the default mode network) and amygdala (Dutta et al., 2014; Kaiser et al., 2015; Northoff, 2016), though these findings have not been entirely consistent. For example, in a group of depressed adolescents, Connolly and colleagues found evidence for increased mPFC (in particular sgACC)-amygdala connectivity, (Connolly et al., 2013) suggesting that age of MDD (or age of onset) may be an important factor in determining the default connectivity of these regions.

Some additional studies indicate that resting state functional connectivity between the anterior cingulate cortex (ACC), lateral PFC and the limbic regions including the amygdala is decreased in MDD (Anand et al., 2005b; Tang et al., 2013). Another study examining amygdala prefrontal connectivity during rest found that individual differences in MDD severity was associated with amygdalar-prefrontal hypoconnectivity across a variety of regions including bilateral dorsolateral PFC, anterior cingulate and anterior insula (Satterthwaite et al., 2015a). Similar findings have been reported for depressed individuals high on rumination (Yoshimura et al., 2010). Other studies have documented decreased intrinsic corticolimbic connectivity, including between the pregenual ACC and the thalamus, amygdala, and pallidostriatum, in depressive and bipolar mood disorders (Anand et al., 2009).

### Structure

A meta-analysis of Voxel-based analysis of DTI studies of adult patients with MDD identified decreased fractional anisotropy in the white matter bundles connecting the PFC amygdala (Liao et al., 2013)—the primary white matter pathway being the uncinate fasciculus. While this finding appears to be relatively consistent in adults (Steele et al., 2005; Carballedo et al., 2011; Murphy et al., 2012; Zhang et al., 2012; de Kwaasteniet et al., 2013) and in adolescents (Cullen et al., 2010; Aghajani et al., 2014; LeWinn et al., 2014), these differences have generally not been found in individuals at-risk for MDD (i.e., with a family history; Frodl et al., 2012; Keedwell et al., 2012; for a recent review, see Bracht et al., 2015b). Using graph theory approaches, Singh et al. (2013) have further found that depressed patients had evidence for higher “betweenness” than healthy controls in the amygdala and OFC—indicating that more short paths crossed through those nodes in depressed patients than healthy controls.

Altogether, there appears to be white matter abnormalities in PFC-amygdala pathways in patients currently with MDD—regardless of whether they are an adult or adolescent. However, this abnormality does not appear to extend prior to disease onset which suggests that this white matter pathway is not necessarily is a prodromal risk-factor for the development of MDD. It would be helpful for future work to combine structural and functional data to control for individual differences in structural abnormalities in examining functional differences between groups (e.g., Oakes et al., 2007).

## Prefrontal-Dorsal Raphe Nucleus Connectivity

The DRN is the site of serotonin synthesis in the brain. The DRN receives direct glutamatergic inputs from the ventromedial PFC (Challis and Berton, 2015). However, the majority of VMPFC projections to the DRN terminate onto local GABAergic interneurons that inhibit 5-HT neurons and gate serotonergic output. In general, these projections impact the excitability of local 5-HT synthesizing neurons within the DRN. A potential role for the DRN in MDD is evidenced by the fact that SSRIs are one of the primary current treatments for MDD. However, given the small size of the DRN, virtually all work mechanistically examining vmPFC-DRN interactions in MDD has done so in animal models thus far.

### Animal Models

Researchers have found direct links between prefrontal dysfunction and susceptibility in learned helplessness paradigms (Maier and Watkins, 2010). Among other neurotransmitter systems, stress activates the DRN. During controllable stress, DRN is engaged less as when uncontrollable stress is delivered as measured by c-Fos expression in 5-HT labeled neurons (Grahn et al., 1999). As such, in the presence of uncontrollable stress, these inhibitory GABAergic projections from the vmPFC to the DRN will typically inhibit DRN synthesis of 5-HT. It has been shown that experimentally engaging these vmPFC projections to the DRN alters the behavioral phenotype in response to uncontrollable stressors and make behaviors in the uncontrollable condition appear “controllable”. Similarly, the opposite has been demonstrated—that inactivation of the rodent vmPFC in response to controllable stressors causes the behavioral phenotype in response to those stressors appear “uncontrollable” (Amat et al., 2005, 2006; Baratta et al., 2009). Interestingly, when excitatory mPFC neurons are directly activated using optogenetics, no effect on MDD-related behavior in either the forced swim or open field tests are observed (Ménard et al., 2015). However, identification and stimulation of specific mPFC efferents to the DRN, FST immobility decreases (Warden et al., 2012).

## Other Circuits

Although not a focus of the review, interactions between the hippocampus and PFC appear to be dysfunctional in MDD—with decreased connectivity between the hippocampus, mPFC and other DMN structures in depressed patients as compared with healthy controls (Kaiser et al., 2015). A central role for the hippocampus and prefrontal-hippocampal interaction in the pathophysiology of MDD is not surprising given a central role for the hippocampus in the regulation of the HPA axis (McEwen, 2001, 2012) as well as it’s site as a key source of neurogenesis in the human brain (Sahay and Hen, 2007). The hippocampus also has glutamatergic projections to the NAc and the amygdala and appears to have an important role in the formation of emotional memories (Krishnan and Nestler, 2010).

Furthermore, there are several neural changes that occur within the hippocampus that result from the social defeat stress model. These include decreased arborization in the hippocampus similar to that of the learned helplessness model (Duman and Monteggia, 2006). Social defeat stress also causes changes in brain derived neurotrophic factor (BDNF) in the hippocampus, and decreased cyclic-AMP-response-element-binding-protein (CREB) activity in the hippocampus. Changes in both of these systems appears to be tied to changes in stress regulation and hippocampal neurogenesis (Duman and Monteggia, 2006; Krishnan and Nestler, 2008). Changes in hippocampal structure in rodent models of MDD are paralleled by consistent findings in human depressed patients of decreased hippocampal size and gray matter density (Singh and Gotlib, 2014) and that the number of depressive episodes in humans is negatively correlated with hippocampal size, further implicating the hippocampus in MDD course (Videbech and Ravnkilde, 2004). Many of the cellular changes in the hippocampus are normalized with treatment in these rodent models. Continued examination of PFC-hippocampal interaction in humans with MDD, both at rest and in response to specific stimuli will be an important area going forward.

## Future Directions

Both nonhuman and human work points to dysfunction in cortical-subcortical circuits in individuals suffering from MDD. However, there are several avenues which must be examined to further the specificity and generalizability of the findings reviewed. For example, a recent study using structural scans across nearly 16,000 patients of differing diagnoses found that structural abnormalities were fairly common across these disorders. In particular, across psychiatric diagnoses there was a decrease of structural integrity within the neural network that included the anterior insula and dACC. It was hypothesized that this finding may relate to executive function deficits observed across a variety of diagnoses (Goodkind et al., 2015). Furthermore, using resting-state fMRI, Oathes et al. (2015) found that a single conceptual model—such as categorical diagnoses incompletely captures the neural presentation of psychopathology. In particular, they argued that their data support that an *additive* model best captures abnormal neural patterns in patients with anxiety and MDD (Oathes et al., 2015)—suggesting severity may be a better way to categorize the nosology than diagnostic criteria or symptom dimensions. These findings, along with the high comorbidity between Axis I diagnoses as well as the use of SSRIs to treat many of these so-called distinct disorders raises the question of specificity—how specific and which of these abnormalities are unique to MDD? The RDoC model attempts to address this issue, although there are few animal models that solely address a single dimension. Emerging work, using support vector machine and classification learning has the potential to greatly enhance the specificity and parsing of networks as a function of psychopathology (Sacchet et al., 2015).

Many of the task-based fMRI studies have used faces, financial rewards or static images to induce affective states. These stimuli have significant value in their ease of use and standardization. However, the magnitude of emotion elicited in these paradigms can be questioned and it is unclear how well they model real-world emotional reactivity and regulation. In addition, in studies that have used emotional faces, without concurrent measurement of autonomic nervous system activity, it is difficult to know whether neural responses to these stimuli are representing the perception of emotion in another individual or in the experience of the emotion. As such, it may be helpful to use more basic stimuli (e.g., odors, juice rewards; McCabe et al., 2009, 2012) or more complex, personally relevant stimuli (e.g., autobiographical memories; Young et al., 2016). Some of these personally relevant stimuli may be less well-standardized than other stimulus options, but may have the virtue of more effectively inducing emotion. This could increase the variance of affect induced—both within and across participants—and lead to more robust tests to examine brain-behavior associations.

Furthermore, as briefly mentioned earlier, performing analyses that integrate structure and function will be important going forward. Postmortem analyses, *in vivo* MRI as well as rodent studies have shown that MDD impacts PFC (Rajkowska, 2000; Rajkowska et al., 2005), amygdala (Krishnan and Nestler, 2008) and hippocampal structure (Stockmeier et al., 2004). As such, controlling for changes in the jacobian determinant (Avants and Gee, 2004), fractional anisotropy or gray matter probability while examining fMRI connectivity maps may be an helpful approach to dissociate effects of the disease course on structure and function (e.g., Heller et al., 2013).

As opposed to relying on a single snapshot self-report to associate with neural markers, research exploiting mobile technology to examine specific symptoms will also be critical. In particular using cellular phone-based ecological momentary assessment (EMA) can circumvent some of these potential shortcomings by capturing real-world emotion as it is experienced (Kaplan and Stone, 2013). These methods allow individuals to respond to text messages prompts longitudinally over days and assess their current emotional functioning. All EMA studies to date assess individuals functioning at baseline (in other words without an explicit stimulus). One new approach is in the use of EMA is to have participants play real-world tasks and games designed to induce affect (Rutledge et al., 2014; Heller et al., 2015). These types of tasks permit a level of experimental control that has not been incorporated in prior EMA designs of psychopathology and can allow researchers to examine the time course of emotional experiences as depressed patients and healthy controls go about their daily lives.

In sum, MDD is a debilitating disorder that is likely subserved by dysfunction is specific neural circuits (Ressler and Mayberg, 2007). Integrating functional and structural neuroimaging with animal models has begun to provide us with a better understanding of the basic science of MDD, and which neural circuits may be dysfunctional abnormal. Continued investigation in this vein will hopefully begin to reveal novel avenues for treatment as well as improve the identification of those as risk.

## Author Contributions

ASH wrote and edited the review article.

## Conflict of Interest Statement

The author declares that the research was conducted in the absence of any commercial or financial relationships that could be construed as a potential conflict of interest.
